# Loss of Abdominal Muscle in *Pitx2* Mutants Associated with Altered Axial Specification of Lateral Plate Mesoderm

**DOI:** 10.1371/journal.pone.0042228

**Published:** 2012-07-31

**Authors:** Diana Eng, Hsiao-Yen Ma, Jun Xu, Hung-Ping Shih, Michael K. Gross, Chrissa Kiouss

**Affiliations:** 1 Department of Pharmaceutical Sciences, College of Pharmacy, Oregon State University, Corvallis, Oregon, United States of America; 2 Department of Pediatrics, Department of Cellular and Molecular Medicine, University of California San Diego, La Jolla, California, United States of America; Ecole Normale Supérieure de Lyon, France

## Abstract

Sequence specific transcription factors (SSTFs) combinatorially define cell types during development by forming recursively linked network kernels. Pitx2 expression begins during gastrulation, together with *Hox* genes, and becomes localized to the abdominal lateral plate mesoderm (LPM) before the onset of myogenesis in somites. The somatopleure of Pitx2 null embryos begins to grow abnormally outward before muscle regulatory factors (MRFs) or Pitx2 begin expression in the dermomyotome/myotome. Abdominal somites become deformed and stunted as they elongate into the mutant body wall, but maintain normal MRF expression domains. Subsequent loss of abdominal muscles is therefore not due to defects in specification, determination, or commitment of the myogenic lineage. Microarray analysis was used to identify SSTF families whose expression levels change in E10.5 interlimb body wall biopsies. All Hox9-11 paralogs had lower RNA levels in mutants, whereas genes expressed selectively in the hypaxial dermomyotome/myotome and sclerotome had higher RNA levels in mutants. *In situ* hybridization analyses indicate that *Hox* gene expression was reduced in parts of the LPM and intermediate mesoderm of mutants. Chromatin occupancy studies conducted on E10.5 interlimb body wall biopsies showed that Pitx2 protein occupied chromatin sites containing conserved bicoid core motifs in the vicinity of *Hox 9-11* and MRF genes. Taken together, the data indicate that Pitx2 protein in LPM cells acts, presumably in combination with other SSTFs, to repress gene expression, that are normally expressed in physically adjoining cell types. Pitx2 thereby prevents cells in the interlimb LPM from adopting the stable network kernels that define sclerotomal, dermomyotomal, or myotomal mesenchymal cell types. This mechanism may be viewed either as lineage restriction or specification.

## Introduction

Mesodermal cells are formed and positioned along the anterior-posterior (AP) axis during gastrulation. At each successive axial level, the earlier ingressing cells will become the LPM whereas the later ingressing cells will become the more medial presomitic, or paraxial, mesoderm. Somites formed by segmentation of the paraxial mesoderm, are further divided into the outer dermomyotome/myotome and inner sclerotome [1]. The dermomyotomes/myotomes give rise to all skeletal muscle precursors. Cells from the dorsomedial lip of the dermomyotome give rise to epaxial muscles, whereas cells from the ventrolateral lip of the dermomyotome give rise to hypaxial muscles [2]. The LPM is subdivided into an outer, more lateral somatopleuric, and inner, more medial splanchnopleuric mesenchyme, which combine respectively, with the surface ectoderm or endoderm to give rise to the somatopleure (SMP) and splanchnopleure (SPP). The SMP forms an outer mesenchymal layer that resides under the epithelial ectoderm, and together with it forms the body wall. Hypaxial muscle progenitor cells are specified in the dermomyotome, well before these move into the SMP. Muscles of the abdominal wall also derive from somites [3]. The SMP cells at abdominal levels delaminate and migrate individually, and the muscle progenitor cells, at least initially, remain locked within the dermomyotomal epithelia as they grow and extend ventrally into the SMP (abdominal wall). The molecular specifications, of both somites and SMPs, differ between axial levels. Consequently, the interactions between somites and SMP produce different outcomes, in terms of muscle development, at each axial level.

The complex interactions between genes and their associated regulatory factors can be modeled with gene regulatory networks (GRNs) [4]. These networks consist of genes (nodes) and functional relationships (links) they have with other genes that they regulate or regulate them. Within a specific developmental domain, there are evolutionarily conserved combinations of nodes and links creating the “kernels.” These kernels often consist of SSTF nodes, which can act on other nodes or themselves to stabilize the developmental state of the cell, often through cis-regulatory modules (CRMs) of each gene involved. This self-stabilization of the nodes within each kernel defines a recursively-linked network. When a single node within a kernel is disrupted, the function of the kernel as a whole is altered and is likely to cause a significant change in the body part in which the specific cell type was required. One notable feature of using kernels to define a cell types, is that the nodes within the kernels are not restricted solely to that kernel, but can be involved in numerous different kernels that make up an organism’s GRN.

Pitx2 is essential for proper organogenesis and myogenesis during mouse development [5]–[8]. Pitx2 is involved in many developmental kernels, as the first branchial arch (BA)-derived structures, abdominal wall, and internal organs are particularly strongly affected. Muscles within the first BA fail to develop, while those in the adjoining second BA become malformed. The early first BA defects result from a specification defect in early gastrulation [9]. This is consistent with the rostral bilateral stripe of Pitx2 expressing cells that is first observed in gastrulae and which gradually changes into the first BA as development proceeds [10], [11]. A second, more posterior, bilaterally asymmetric, zone of Pitx2 expression is also observed in the vertebrate gastrulae [10], [11]. This zone gradually changes into the body wall/SMP and SPP as development proceeds. Pitx2-dependent changes to the abdominal body wall are subtle at E9.5 [8]. They become severe and obvious by E10.5 [5], [6]. Similar to the first BA, muscles fail to develop in this region, and form abnormally in closely adjoining regions ([9]; this report). A third major zone of Pitx2 expression is the muscle lineage [9], [12]. The Pitx2^LacZ^ knock-in allele is expressed in most, if not all, muscle anlagen at E12.5 and muscles in adults [13]. Pitx2 gene expression is first observed in somites at forelimb levels at E10 [14]. Pitx2 expression dynamically expands to more rostral and caudal somites during the E10–E10.5 interval, and is observed robustly in all somites by E11.5 [13], [14]. The expansion of Pitx2 expression to more rostral somites is closely correlated with the rostral expansion of Pitx2-expressing SMP. Thus, the onset of Pitx2 expression in somitic cells is juxtaposed with the Pitx2-expressing SMP [13]. Myogenic cells and recognizable muscle anlagen develop, albeit somewhat abnormally, in the skeletal muscles of Pitx2 mutants. This occurs despite the fact that Pitx2 is expressed at all stages of the myogenic progression, from migratory Pax3^+^/Lbx1^+^ (at limb levels), to Myod1^+^ or Myf5^+^ determined myoblasts to Myogenin^+^ committed myocytes. The restriction of Myf5, Myod1 and Myogenin expression to Pitx2 expressing cells in the limb muscle anlagen, suggests Pitx2 is engaged in recursive interactions with the MRF genes in the context of several network kernels that establish a set of interacting cellular states that locally control muscle fiber growth. Pitx2 is also involved in several network kernels that establish the state of myoblast adhesion, migration and motility [15].

Network kernels represent a recursively-linked, rather than sequential, hierarchical regulatory model of development [16], [17]. Genes involved in establishing the axially restricted *Pitx2* expression domains during early gastrulation would also receive recursive input from *Pitx2.* Axial specification along the trunk is controlled by sequential deployment of *Hox* genes in a rostral to caudal direction. Nested sets of *Hox* genes are expressed, in both ectoderm and mesoderm, in a staggered fashion along the body axis [18]. Expression of each *Hox* gene suppresses the previous, in a more rostral specification. This principle of posterior dominance of *Hox* genes is well established in mammals [19]. *Hox* genes are well known for their roles in axial specification during gastrulation, and those *Hox* genes that are critical for specifying first BA and abdomen could reasonably be expected to receive recursive input from *Pitx2.*

We are interested in discovering the architecture of Pitx2-dependent network kernels. We hypothesize that Pitx2 is involved in the gene network of the LPM that specifies the abdominal muscles. We expect that a variety of Pitx2-dependent network kernels functionally define cell types (states) within the first BA, LPM, and muscle anlagen. We therefore must gather molecular interaction data between the Pitx2 protein and CRMs in other SSTF genes to build a sufficiently detailed network model that allows the particular architectures of each recursive network kernel to be recognized. SSTF genes that change their expression in a *Pitx2*-dependent manner at relevant times and places of development are therefore of particular interest. We have previously used high-throughput expression analyses to compare the expression levels of all SSTF genes in the body wall/somites of normal and *Pitx2* mutant embryos at E10.5 [20]. These studies, which focused on *Pitx2*-dependent T-box genes, also identified many other SSTF genes that changed their expression levels in a *Pitx2*-dependent manner. These included several Hox genes and Myf5, which seemed directly relevant to abdominal wall development. The “genetic” SSTF target genes identified by the microarrays could involve direct physical interactions between the Pitx2 protein and a CRM in target genes. Here, we report the abdominal muscle phenotype of Pitx2 mutants and examine Pitx2 protein occupancy at evolutionarily conserved bicoid core motifs in the MRF and Hox loci in embryonic body wall biopsies. Pitx2 protein physically occupies a large fraction of these sites in the chromatin of the embryonic body wall. The data demonstrate that physical interactions between Pitx2 protein and the regulated “genetic” target genes occur *in vivo*.

## Results

A detailed analysis of the body wall muscle phenotype of late stage *Pitx2* mutants has, to our knowledge, not yet been reported. Whole mount X-gal stains of heterozygote (Pitx2^LacZ/+^) and homozygote (Pitx2^LacZ/LacZ^) mice were therefore compared at E14.5, the latest stage that mutant embryos could be effectively obtained. The severe bending of mutant bodies to their right at abdominal levels made it difficult to compare the muscle anlagen. However, by focusing on attachment points and consulting a comparative anatomy laboratory textbook, we identified the anlagen in E14.5 mice that appear to be present, absent, or modified in mutants. The attachment points of each muscle are indicated by sets of colored dots that should be compared between the corresponding heterozygote and mutant embryos. The muscles of the body wall can be roughly divided into two categories. The trunk axial musculature connects non-limb bones, or fascia, with each other and consists of both abdominal wall and non-abdominal muscles (vertebral/epaxial). The limb-suspension musculature consists of muscles that connect limb bones with axial fascia or bones. Only a subset connects to abdominal fascia or extends through the abdominal region.

### Pitx2-Dependent Defects in the Abdominal Axial Trunk Musculature

**Figure 1:**
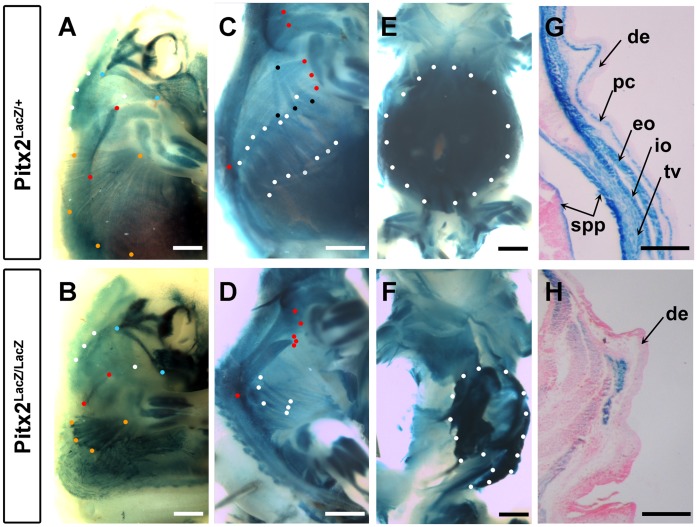
Body Wall Muscle Defects in *Pitx2* Mutants. Whole-mount X-gal staining to compare nascent abdominal and limb suspension musculature at E14.5 in heterozygote, Pitx2^+/LacZ^ (**A,** **C,** **E,** **G**) and mutant, Pitx2^LacZ/LacZ^ (**B,** **D,** **F,** **H**) mouse fetuses. Mutant fetus has a sharp rightward kink in the body axis that was straightened somewhat to allow visualization. Right sides are shown because they are less malformed than left sides. Colored dots indicate approximate location of the attachment points from which were used to identify muscle anlagen. (**A,** **B**) Skinned fetuses show the most superficial muscles beneath the skin. Spinotrapezius (red dots) and latissimus dorsi (orange dots) were truncated at the abdominal attachment end and had faulty texture. Acromiotrapezius (white dots) and levator scapulae ventralis (blue dots) were of normal length but faulty texture. Scale bar, 1 mm. (**C,** **D**) Muscles identified in outer layer have been dissected away. Serratus dorsalis (black dots) was not detected in the mutant or was displaced (white dots). Two straps of sacrospinalis (red dots) appeared to reach the vertebral column, but were abnormally shortened and of incorrect texture. External oblique (white dots) muscle anlagen in heterozygotes are either absent or severely truncated on their abdominal end. Scale bar, 1 mm. (**E,** **H**) Ventral view of the abdominal wall shows complete collapse of musculature (white dots). Scale bar, 1 mm. (**G,** **H**) Frontal sections through the abdomen of X-gal stained and paraffin embedded fetuses, counterstained with eosin/hematoxylin. The left side of the embryo is shown in both panels. The residual muscle fragment cannot be definitively identified. Scale bar, 200 µm. **de**, dermis; **pc**, panniculus carnosus; **spp**, spachopleural mesenchyme; **eo**, external oblique; **io**, internal oblique; **tv**, transversus.

The abdominal muscles consist of three muscle sheets within the lateral body wall and one long cord-like muscle, the rectus abdominus, which runs along the ventral midline. These four muscles were stained by X-gal in heterozygotes but were difficult to photograph, even in skinned embryos, because the non-muscular layers derived from SMP and SPP also stain with X-gal (Fig. 1G). The open abdominal body wall of mutants was also deeply stained by X-gal, but this was an amorphous blue staining in which muscle anlagen were not evident (Fig. 1F, H). The outer abdominal muscle sheet, or external oblique muscle was absent on the left side and appeared to consist of three vestigial straps on the right side (Fig. 1C, D; white dots). The three vestigial straps of muscle retained on the right side may also be interpreted as displaced versions of the serratus dorsalis. No evidence could be obtained to support the existence of possible vestigial forms of the internal oblique muscle (middle sheet), the transversus muscle (deep sheet), and the rectus abdominus.

The non-abdominal axial musculature of the trunk consists of deep back, superficial back, and intercostal muscles. Deep back musculature consists of the many small muscles that attach the vertebrae to each other and includes more superficial muscles that are located outside of the vertebrae and ribs but do not connect to limb bones. The vertebral arches have not closed dorsally at E14.5. The anlagen for the deep back muscles that connect the vertebrae are just beginning to split off the dorsomedial edge of the expanding myotome at this stage [21] and could therefore not be specifically delineated in our whole mount X-gal stains. Dorsal views showed similar levels and distribution of intervertebral staining intensity in mutant and heterozygote embryos. No apparent differences were observed in serial paraffin sections either (data not shown).

The external and internal intercostal muscles slant in opposite directions and connect the ribs. Although they are hypaxial muscles by innervation, they embryologically derive from the dorsomedial myotome and therefore should be grouped with the epaxial deep back muscles as primaxial muscles [22]. Mutant embryos developed both layers of intercostal muscles, but these appeared reduced and distorted (data not shown). The superficial back muscles could be examined by dissecting away the skin and suspension muscles of X-gal stained E14.5 embryos (Fig. 1C, D). A dark longitudinal blue muscle, the sacrospinalis, is observed dorsolateral to the neural tube along the rostrocaudal axis of heterozygotes. It has a region of intense staining just above the last ribs and appears to split into two components at thoracic levels. One component consists of a strap-like muscle that tracks along the vertebral column toward the skull and one component consists of a muscle that fans out over the ribs at thoracic levels (Fig. 1C; red dots). The region of intense staining above the last ribs appears to persist in mutants, and a reduced strap of muscle extends along the vertebral column toward the skull. However, the muscle that fans out over the ribs appears to be replaced by a strap that does not fan out over the ribs (Fig. 1D; red dots). The sacrospinalis is reduced and can no longer make proper anterior connections. Another superficial back muscle, the serratus dorsalis connects the lateral aspects of the ribs to an aponeurosis. Three X-gal stained bands of muscle that interdigitate with the external oblique attachment points on the ribs and extend dorsally and rostrally at an oblique, were observed in heterozygotes (Fig. 1C; black dots). They were not observed at this location in mutants. However, three bands extending in the same direction from the ribs were observed at a more posterior location (Fig. 1D; white dots). These may be either vestigial external oblique muscles with abnormally dorsal attachment points, or altered serratus dorsalis without an appropriate dorsal aponeurosis connection point.

### Pitx2-Dependent Defects in the Limb Suspension Musculature

Lateral aspects of the forelimb are connected to vertebrae and dorsal fascia by the trapezius, lattissimus dorsi and levator scapulae ventralis. Medial aspects of the forelimb are connected to the ribs, sternum and vertebrae by the pectoralis, xiphihumeralis, serratus ventralis, and rhomboidius. Clavobrachialis connects the clavicle to the forelimb bone. Lateral aspects of the hindlimb are connected to the vertebrae and pelvis by the gluteal, tenuissimus, and caudofemoralis muscles. Medial aspects of the hindlimb are attached to the trunk by the iliopsoas.

The spinotrapezius of heterozygotes was seen just under the skin as a blue stripe that extended caudally from the spine of the scapula along the ventral edge of the vertebral column (Fig. 1A; red dots). A similar stripe was observed in mutants, but the stripe did not extend as far caudally (Fig. 1B; red dots). The acromiotrapezius attaches the scapular spine with more rostral vertebrae and was observed as a superficial blue fan with neat striations in heterozygotes (Fig. 1A; white dots). A similar blue fan was observed in mutants, but it lacked the smooth striation of the normal muscle (Fig. 1B; white dots). The levator scapulae ventralis attaches the scapula to the atlas and occipital bone of the skull. It was seen as a narrow blue strap with fine striations in heterozygotes (Fig. 1A; blue dots). A condensed blue cord with no visible striations was observed in mutants (Fig. 1B; blue dots). The latissimus dorsi connects the posterior aspect of the humerus with the body wall fascia and was seen as a blue fan in the most superficial view of X-gal stained heterozygotes (Fig. 1A; orange dots). This muscle attached to the humerus in mutants but was stunted and knotty in appearance as it fanned out towards the body wall fascia (Fig. 1B; orange dots). Other suspension muscles of the limb were also stained by X-gal in heterozygotes, and corresponding patterns of muscle anlagen were observed in mutants (Fig. 1E, F; data not shown). The contrast between the smooth texture and lighter staining of the latissimus dorsi in heterozygotes and the knotty texture and deeper staining in mutants exemplifies a qualitative difference that appears in all the muscles examined. This is likely to be caused directly by *Pitx2* functions in the kernel defining the muscle cell lineage. The absence or stunting of muscles occurred only in those muscles that had both or one attachment point in the abdominal region. Thus, *Pitx2*-dependent loss or stunting of muscle occurs secondarily due to loss of the abdominal body wall.

### Onset of Body Wall Phenotype

**Figure 2:**
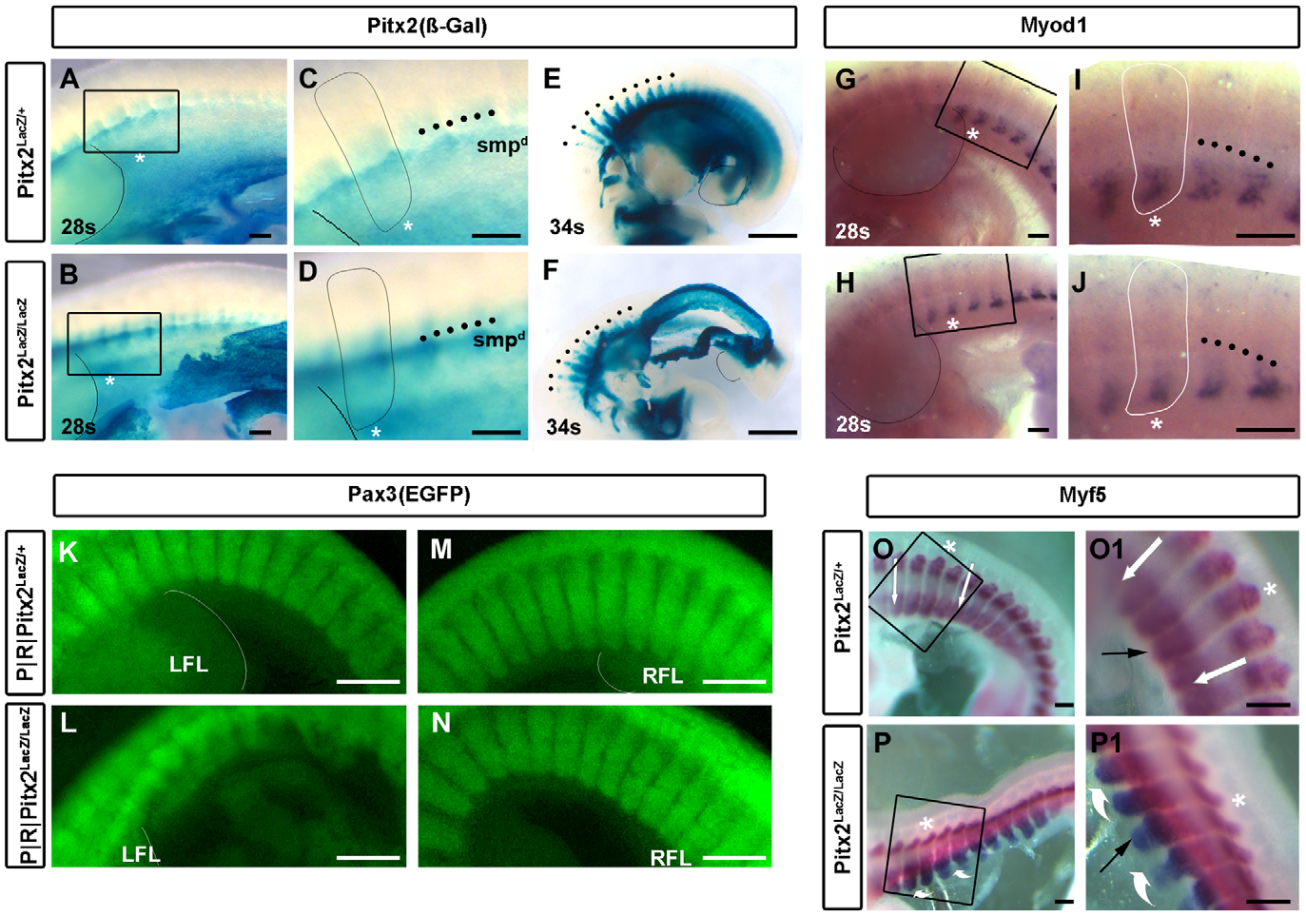
Somitic MRF Expression Precedes the Onset of Somitic Pitx2 Expression. (**A–D**) A lateral stripe of X-gal staining the dorsal somatopleure (smp^d^; black dots), forms from the X-gal stained ventral somaopleure at the posterior forelimb level prior to the 28 somite (E10.5) stage shown here and extends in both the rostral and caudal direction. Underlying somites become X-gal positive as the smp^d^ forms over them. This happens equivalently in mutants and heterozygotes. The second somite behind the left forelimb (asterisk) is enlarged and outlined. Note that somitic X-gal staining is not concentrated at the hypaxial tip. Scale bar, 200 µm. (**E,** **F**) X-gal staining of late E10.5 embryos is similar in the epaxial myotomes (black dots), but large deformities already exist in the body walls of mutants. Scale bar, 1 mm. (**G–J**) Expression domains of Myod1 in hypaxial myotomes of interlimb somites are not altered. The second somite behind the left forelimb (asterisk) is enlarged and outlined, for direct comparison with A–D panels. Note the modest deformity imposed by the outward turn of the body wall. Scale bar, 200 µm. (**K–N**) Pax3^CRE^|ROSA^EGFP^ was used to visualize the entire dermomyotome lineage on the left (**K,** **L**) and right (**M,** **N**) side of E10.5 mice. Note the distortion of somite structure behind the left, but not right, forelimb. Scale bar, 400 µm. (**O,** **O1,** **P,** **P1**) Myf5 expression domains in mutant somites show no apparent difference. The fourth somite behind the forelimb is identified by an asterisk. Black arrows point to the ventral edge of the hypaxial compartment, white arrow and swooshes indicate dorsal to ventral trajectories. Scale bar, 200 µm.

**Figure 3:**
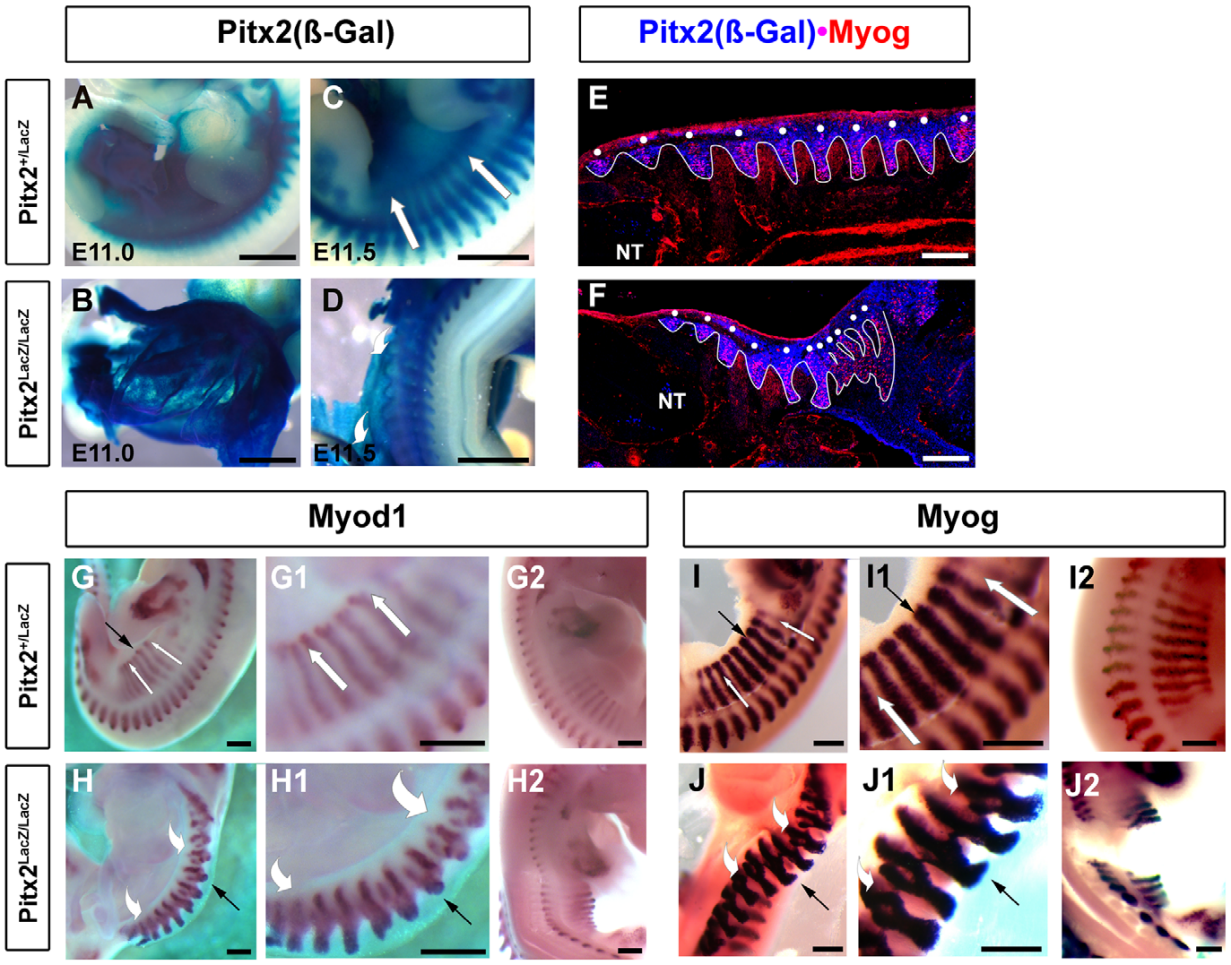
Maintenance of MRF Expression. (**A–D**) X-gal staining of E11.5 embryos reveals the persistence of ß-Gal protein in the SMP and somites**.** Note that hypaxial somites are now buried in ventral abdominal SMP. Note the thinning of ventral body wall, the rightward kink in the body axis, and the abnormal upward turn of the left body wall in mutants (white arrows vs. swooshes). Scale bar, 1 mm. (**E–F**) Double labeling immunohistochemistry of sagittal sections of E11.5 heterozygote and mutant Pitx2 mice indicated colocalization of ß-Gal(Pitx2) with Myogenin in somites, as they get embedded into the SMP. Note the misshaped mutant posterior abdominal somites. Scale bar, 200 µm. (**G–H2**) Whole mount in situ hybridization of E11.5 embryos with Myod1 probe. (**G,** **G1,** **H,** **H1**) Left body walls; abnormal outward turn of body wall is indicated by white arrows and swooshes. Black arrows indicate the ventral tips of the hypaxial abdominal extensions of the fourth somite behind the forelimb. Note that normal internal bifurcations have become visible in mutants because of somite distortion. Note that the tips of the somite extensions are stunted and do not extend to the extreme edge of the upturned body wall. Scale bar, 500 µm. (**G2,** **H2**) Right body walls; Embryos need to be pinned to straighten the rightward kink in the body axis so that the anatomy of right side somites can be examined. The right body wall also turns upward slightly during this pinning process. Note that the apparent distance between the epaxial and hypaxial expression domains appears to widen in mutants, likely due to pile up of dermatome over the central somitic region. Expression domains are similar and stunting near the ventral edge of body wall is also observed. (**I–J2**) Whole mount in situ hybridization of E11.5 embryos with myogenin probe. Scale bar, 500 µm. (**I,** **I1,** **J,** **J1**) Left body walls; descriptions are as for Myod1 above. (**I2,** **J2**) Right body walls; descriptions are as for Myod1 above.

The initially subtle, morphological defects associated with defective abdominal wall closure have been well quantified at E9.5 [8]. The left body wall of Pitx2 mutants begins to bend outward rather than inward at this stage. Pitx2 is robustly expressed in the body wall, but no expression is observed in any presomitic mesoderm or somites at this early stage [13]. The outward bend was accompanied by abnormal rightward bending of the main body axis (Fig. 2C–F), became more severe as development proceeded (Fig. 1–3), and prevented the body wall closure. This may cause the rightward bend of the body axis secondarily due to the absence of physical counterpull by a left body wall. Alternatively, the left body wall may grow too quickly longitudinally because it is not growing ventrally.

### Expression of the Pitx2 Gene Does not Require Pitx2 Function

The onset of Pitx2^LacZ^ expression in mutant somites is still tightly correlated with juxtaposition of Pitx2^+^ SMP^d^ (Fig. 2A, B). The onset of Pitx2 gene expression in somites is therefore *Pitx2*-independent. The onset and maturation of the Pitx2^LacZ^ expression pattern within individual somites appears normal in mutants. This is best seen by comparing the Pitx2^LacZ^ expression in successive somites, proceeding from caudal to rostral at E10 (Fig. 2A–D) and E10.5 (Fig. 2E, F). Pitx2 expression in each somite began at the rostral and caudal edges at the level covered by the SMP^d^ stripe (Fig. 2C, D). Expression expanded along the somite edges in both dorsal and ventral directions. Expression between the two edges lagged somewhat behind, but expanded in a similar fashion (Fig. 2E, F). Cross sections at forelimb limb levels at E10.5 indicate that Pitx2 expression expands mainly into Pax3^+^ cells located on the medial surface of the dermomyotome and to a lesser extent on the lateral surface of the dermomyotome, so that a central Pax3^+^ region remains free of Pitx2 expression [13]. Pitx2^LacZ^ expression in the most dorsal aspect of the myotomes (black dots) can be seen in whole mount X-gal stains at E10.5, because the more superficial dorsal dermomyotomes lack Pitx2 (Fig. 2E, F).

### Onset of Somite Phenotype

Pax3 expression in the paraxial mesoderm initiates as somites formed from the segmental plate become restricted to the dermomyotome prior to E9.5, and begins to show higher levels in the ventrolateral dermomyotome at E9.5 [23]–[25]. The first embryonic expression of Myod1 occurs in the ventral tip of interlimb dermomyotome at E9.5–10 [26], [27]. Neither of these dermomyotome markers showed obvious deficiencies in their expression level in the abdominal somites of *Pitx2* mutants at E10.5 (Fig. 2G–J; K–N). Dermomyotomes, just caudal to the forelimbs extend their ventrolateral lips into the abdominal body wall as early as E9.5, prior to the onset of Pitx2 expression in the paraxial mesoderm. By E10.5, the abdominal somites expressed Myod1 robustly (Fig. 2G, I), even though little or no Pitx2 expression was observed at this location (Fig. 2A, C). Morphological differences in the shape of the ventral extensions were observed in both Myod1 RNA (Fig. 2G–J) and Pax3 lineage trace (Fig. 2K–N) staining. These differences were observed on the left side, and indicated that the dermomyotomal components of the abdominal somite extensions had entered the abnormally turned body wall at E10.5. The degree of body wall turning differed significantly between mutant littermates, indicating a variable penetrance of this phenotype (compare Fig. 2L and H).

Myf5 and Myf6 RNA expression begins at the dorsal and ventral myotome-forming lips of the dermomyotome respectively, soon after somites come off the segmental plate. Both RNAs are expressed throughout the myotome at interlimb levels by E9.5. Myf5 expression extends slightly further ventrally than Myf6 [28]. The abdominal extensions continue to actively express Myf5 at E10.5 [29] and E11.5 [30]. Loss of *Pitx2* function had an effect on the Myf5 expression levels at E10.5 in the dorsal aspect of the myotome and in the abdominal extensions (Fig. 2O–P1). Quantitative analysis of MRF expression indicated increased expression levels in the abdominal wall biopsies (Fig. S1), which was in accord with the microarray data of Table 1. The outward curl of the ventral half of the Myf5 expression domain on the left side indicated that the hypaxial myotome had also extended into the abnormal body wall by E10.5 (Fig. 2P, P1). *Pitx2* function had little to do with the initiation of Myf5, Pax3, or Myod1 expression in the abdominal somites. This was not surprising, because Pitx2 expression in somites begins at E10.5, as somites just come into contact with the Pitx2^+^ somatopleure (Fig. 2A, C; [13]) and is not likely a part of the network kernel that initially defines muscle progenitors in somites.

**Table 1:** Pitx2 Target Genes in Abdominal Wall.

Gene	Chr	RefSeq ID	FoldΔ	Phenotype	Bibliography	P-value^1^
**Hoxa9**	6	NM_010456	**−1.8±0.1**	Anteriorization of vertebrae L1–L5	[32]	<0.0001
**Hoxb9**	11	NM_008270	**−1.3±0.2**	Rib formation defects	[32]	0.46
**Hoxc9**	15	NM_008272	**−1.2±0.1**	Anteriorization of vertebrae T10–L1	[62]	0.0041
**Hoxd9**	2	NM_013555	**−1.7±0.1**	Anteriorization of vertebrae L3–L5, S2, S4	[63]	0.0041
**Hoxa10**	6	NM_008263	**−2.2±0.2**	Extra rib formation in lumbar to sacral regions	[38], [39]	0.0002
**Hoxc10**	15	NM_010462	**−3.8±0.5**	Extra rib formation in lumbar to sacral regions	[38], [39]	0.0026
**Hoxd10**	2	NM_013554	**−2.1±0.1**	Extra rib formation in lumbar to sacral regions	[38], [39]	0.0138
**Hoxa11**	6	NM_010450	**−2.6±0.0**	Provides sacral and lumbar identity	[38], [39]	0.0001
**Hoxd11**	2	NM_008273	**−1.3±0.0**	Provides sacral and lumbar identity	[38], [39]	0.0022
**Myf5**	10	NM_008656	**2.2±0.3**	Delayed myotome formation, partially formed ribs	[64]	0.16
**Myf6**	10	NM_008657	**1.3±0.2**	Defects in skeletal muscle maturation, rib abnormalities	[65]	0.098
**Myog**	1	NM_031189	**1.3±0.2**	Delayed myogenesis, thoracic skeleton defects	[66]	0.316
**Myod1**	7	NM_010866	**1.2±0.1**	Functional equivalence with Myf5	[67]	0.06
**Pax3**	1	NM_008781	**−1.3±0.3**	Defects in migration of limb muscle precursors	[25]	0.22
**Pax7**	4	NM_011039	**1.2±0.3**	Defects in delamination, migration, proliferation of muscle precursors	[68]	0.85
**Pdgfc**	3	NM_019971	**1.6±0.02**			<0.0001
**Pax1**	2	NM_008780	**1.3±0.07**			0.2
**Scx**	15	NM_198885	**1.5±0.08**			0.046
**Tnc**	4	NM_011607	**3.6±0.5**			0.0002

1 Based on Student’s t-test.

2 Hoxc11 is not represented on the Mouse Genome 2.0 Array.

Abdominal somites were severely deformed one day later at E11.5, in *Pitx2* mutants. Deformation was of an entirely different character on the left and right sides of the body. Somites were bent upward and outward with the left body wall, whereas they became thickened, shortened and compacted within the right body wall, which was sharply kinked to the right at this stage (Fig. 3). Myod1 and Myogenin RNAs at E11.5 are normally expressed in the dorsal myotome and the ventral extension of abdominal somites (Fig. 3E, G, G1, G2, I, I1, I2). However, both domains were slightly further apart, or embedded deeper into the body, in the right body wall of mutants (Fig. 3H2, J2), whereas the ventral extension was curled outward, over the dorsal myotome and dermotome, in the left body wall of mutants (Fig. 3H, H1, J, J1). The left somites of mutants appeared to be abnormally bifurcated. Double labeling immunohistochemistry for antibodies against ß-Gal(Pitx2) and Myogenin in the abdominal somites at E11.5 (Fig. 3E, F) further support the above observations. Pitx2^+^ somites were also Myogenin^+^. The most posterior dorsal somites were not well defined and were irregularly embedded into the SMP (Fig. 3F). The continued expression of the MRFs in the abdominal extensions of mutants at E11.5 indicates that Pitx2 is not required for maintenance of MRF expression in abdominal somite extensions at later stages. *Pitx2* was therefore not required for establishment or maintenance of MRF expression in the abdominal somite extensions. The somite malformations at E11.5 and later abdominal muscle defects therefore do not result from defects in specifying the muscle lineage *per se*. Instead, they appear to result secondarily from severe *Pitx2*-dependent defects of the SMP context into which these somites normally grow. This is consistent with the complete loss of abdominal muscles and the single-ended stunting of suspension muscles at the end that attaches to the abdominal wall. It is also consistent with the phenotypic differences observed, in both somites and muscles, between the left and right body walls. The abdominal extensions of mutants were stunted close to the edge of the left (Fig. 3H, H1, J, J1) and right (Fig. 3H2, J2) body wall.

### Selective Regulation of Hox9, 10, 11 Paralogs in the Abdominal Wall

The molecular events that lead to the deformation of the body wall are not understood. We have used microarrays to compare gene expression in the abdominal body walls of normal and *Pitx2* mutant embryos at E10.5 [20]. Abdominal wall tissue was obtained by cutting across embryos between the limbs and removing the neural tube and viscera. The body wall with some somitic tissue was extracted. Total RNA preparations from three pools of Pitx2^+/+^, three pools of Pitx2^LacZ/+^ and three pools of Pitx2^LacZ/LacZ^ embryos were used to prepare the probes for nine Affymetrix Mouse Genome 430 2.0 gene expression microarrays. This analysis revealed several families of SSTFs regulated by *Pitx2* [20]. Here we present an analysis for *Pitx2*-dependent Hox and MRF genes (Table1).

**Figure 4:**
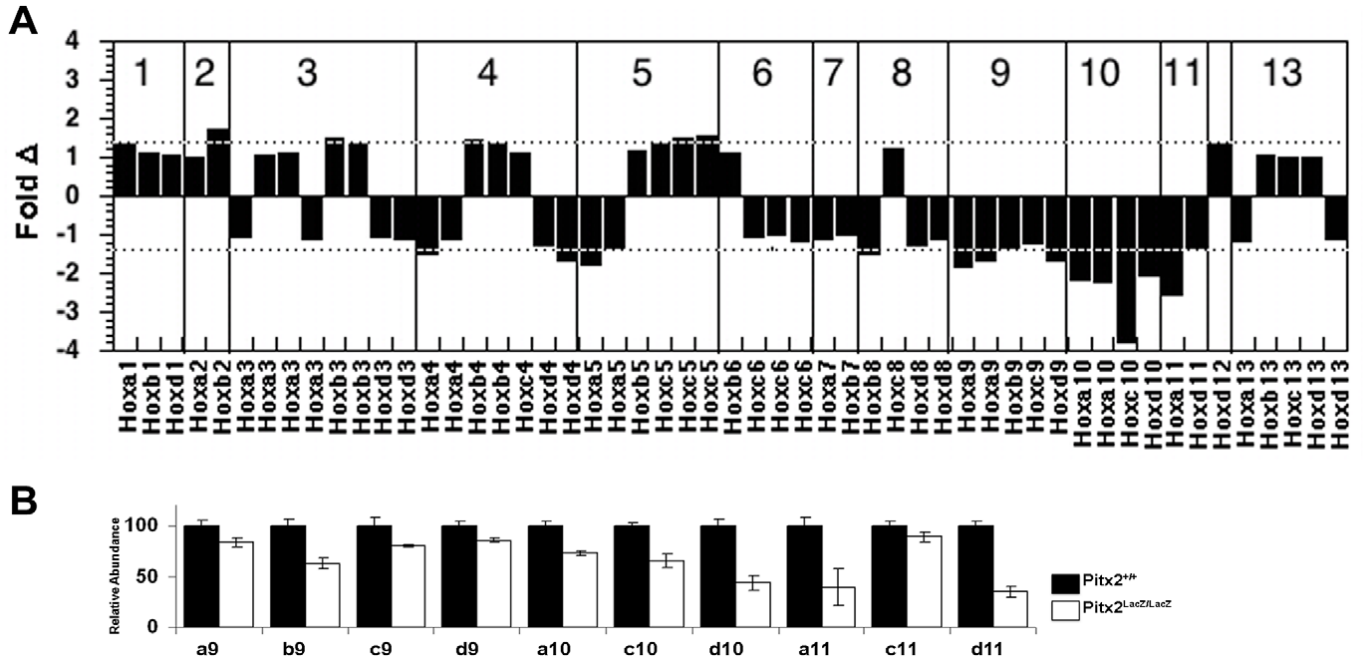
Expression Levels of Hox Genes in E10.5 Abdominal Wall Biopsies. (**A**) Fold changes in Hox expression levels in total RNA from E10.5 interlimb abdominal wall biopsies. The average signals from two biological replicate arrays, each made from pools of several embryos, are compared. Positive fold change indicates higher expression levels in mutants, or genetic repression by *Pitx2*. Negative fold change indicates lower expression levels in mutants, or genetic activation by *Pitx2*. The dashed lines indicate a false discovery rate of 11.5% at the ±1.4 fold threshold, determined by fold scanning analysis for three biological replicates, prior to removing the outlier set. The false discovery rate at this threshold is expected to be lower without the outlier. If multiple probe sets on the arrays monitored the expression of individual genes, then the probe set that produced the highest average signal intensity over all arrays is shown. This is generally the 3¢ most probe set on the transcription unit. (**B**) qPCR measurements on total RNA from E10.5 inter-limb body wall biopsies.

*Hox* gene expression was collectively compared between wild type (WT) and mutants (MUT) by 53 probe sets. Eleven of these (20%) changed more than 1.4 fold. At this fold cut off, only 1% of all probe sets (450 of 45,101) on the array are “regulated” with a false discovery rate of 11.5%, as measured by fold-scanning analysis [31]. The fact that 20% of Hox probe sets were “regulated” at the same threshold indicates that *Pitx2* modulates expression from Hox clusters selectively. One of the three sets of biological replicates considered above was an outlier [20]. If the arrays from this set are not considered, then 18 of the 53 Hox probe sets (34%) changed more than 1.4 fold. Approximately half of these were probe sets for Hox 9, 10, and 11 paralogs. The probe sets with the greatest fold changes among all the Hox probe sets were in this set (Fig. 4A). In addition, all Hox 9, 10, and 11 probe sets registered lower expression levels in mutants (Table1). This was confirmed by qPCR analyses (Fig. 4B). Loss of Hox 9, 10, and 11 paralogs leads to deficits in ribs and in lumbar and sacral vertebrae (Table1). These structures occur at approximately the same axial levels as the abdominal body wall.

### Loss of Small Hox 9, 10, and 11 Expression Domains in Abdominal Body Wall

**Figure 5:**
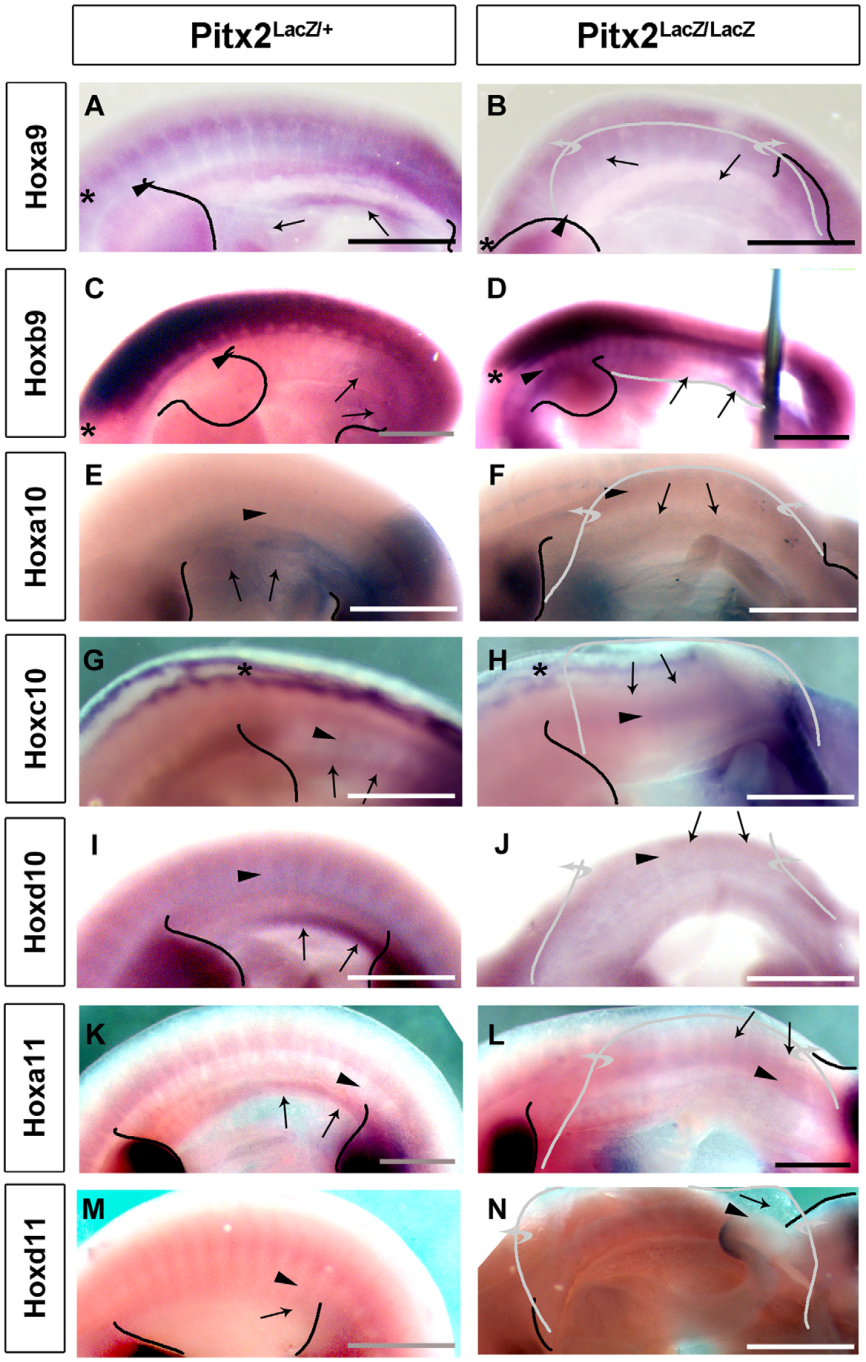
Loss of Abdominal *Hox 9-11* Expression Domains in Pitx2 Mutants. *In situ* hybridization with indicated Hox probes on heterozygote (**A,** **C,** **E,** **G,** **I,** **K,** **M**) and mutant (**B,** **D,** **F,** **H,** **J,** **L,** **N**) E10.5 mice. Left sides are shown, with head to the left. Limb buds are traced in black and the upturned body walls (swooshes) of mutants are traced in grey. The anterior expression boundary in the neuroectoderm/DRG is indicated by an asterisk, except in panels G and H where the asterisk indicates equivalent axial levels at which expression in the dorsal midline converges. Black arrowheads indicate the approximate expected anterior expression boundary in the sclerotome compartment of the paraxial mesoderm. Based on phenotypic considerations, this boundary lies just behind the forelimb, just behind the ribs, and just prior to the hindlimbs for Hox 9, 10, and 11 paralogs, respectively. Black arrows indicate zones of expression in heterozygoetes and equivalent positions in the upturned body walls of mutants, or in the intermediate mesoderm where expression is not observed. Note that expression of Hoxd11 in the caecum is still present in mutants (**N**), but cannot be seen in heterozygotes (**M**) because it is inside the intact body wall. All embryos are positioned with head to the left. Scale bar, 1 mm.

Whole mount *in situ* hybridization (WISH) was used to determine if Hox 9, 10, or 11 expression declines in *Pitx2* mutants, in at least a subset of the structures that normally express Pitx2 (Fig. 5). Expression of Hox9, 10, and 11 paralogs at E10.5 occurs in neuroectoderm, somites, and LPM. Pitx2 expression was readily observed in the most anterior interlimb somites at E10.5 using whole mount analyses (Fig. 2). However, expression of the Hox 9, 10, and 11 paralogs was not apparent in these somites, or indeed in any interlimb somites, in control embryos (Fig. 5). This was not due to a lack of signal, because somitic expression was observed at hindlimb levels, and was often strong in another domain. Published E10.5 whole mount data for the expression profiles of Hoxa9 [32], Hoxb9, Hoxa10, Hoxc10, Hoxa11 [33], and Hoxd11 [34] show that the anterior boundary of somitic expression for these genes lies at the hindlimb level. Hox gene expression begins in the presomitic mesoderm and is generally observed throughout a particular somite at the time it buds off the presomitic mesoderm. However, Hox expression tends to be restricted to the interior sclerotome by the time that myotome/dermomyotome form. Thus, the bone-forming sclerotomes lack Pitx2 expression, while the muscle forming myotome/dermomyotome lacks Hox expression.

Pitx2 expression in the LPM begins several days prior to E10.5 and was still robust in the interlimb region. Hox9, 10, and 11 expression domains were identified in the interlimb body wall at E10.5 (arrows in Fig. 5A, C, E, G, I, K, M). These domains are difficult to visualize because they are in thin layers of tissue (either SPP or SMP). Little is known about Hox expression in the LPM, and from anatomical assignment of these domains is not yet possible. However, it appeared that some of these domains were lost or altered in mutants (arrows in Fig. 5 B, D, F, H, J, L, N). Side by side comparisons of control and mutants were complicated because the left body wall of the mutant (outlined in gray) is abnormally averted (indicated by swooshes). Hox RNA expression was lost from elongated domains (Hoxa9, Hoxd10, Hoxa11) and from broad layered domains (Hoxb9, Hoxa10, Hoxc10, and Hoxd11).

### Pitx2 Protein Occupancy at MRF and Hox Loci in the Abdominal Body Wall

**Figure 6:**
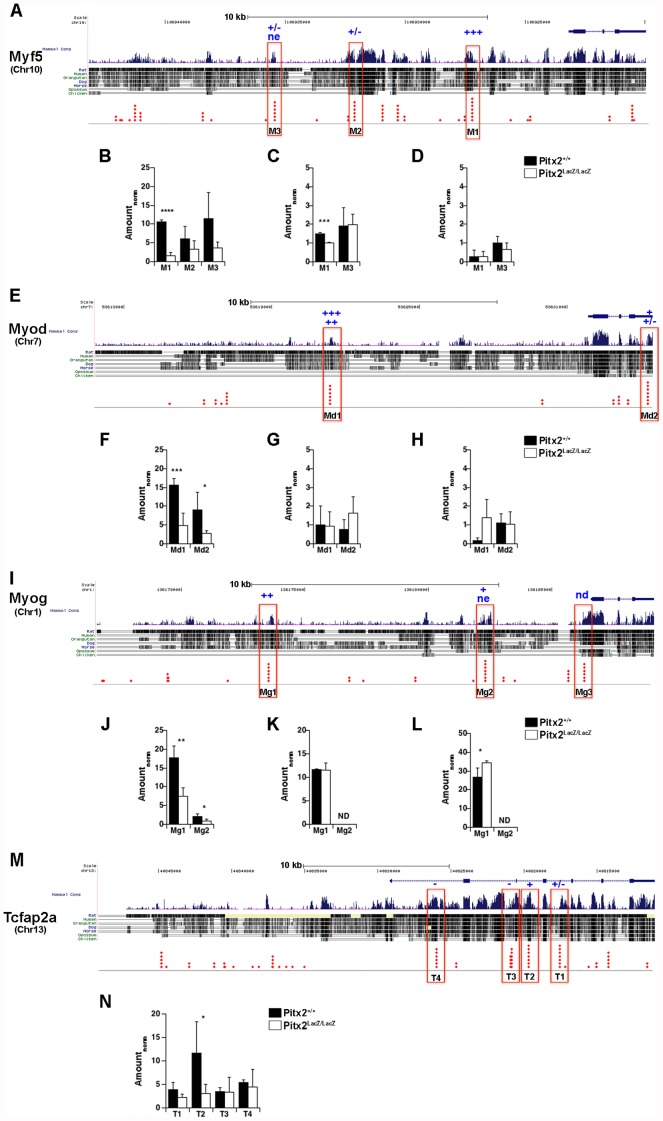
Pitx2 Protein Occupancy at MRF Loci in E10.5 Body Wall Chromatin. The Myf5/Myf6 (**A–D**), Myod1 (**E–H**), Myogenin (**I–L**), and Tcfap2 (**M,** **N**) loci were examined for Pitx2 (**B,** **F,** **J,** **N**), HDAC1 (**C,** **G,** **K**), and HDAC3 (**D,** **H,** **L**) occupancy in sonically sheared chromatin isolated from E10.5 body wall biopsies. PCR amplicons of 70–150 bp (red boxes) were designed around evolutionarily conserved bicoid core motifs. Each red diamond indicates a separate vertebrate species, which contains the bicoid core motif. Bar graphs show the average amount of signal precipitated from wild type (black) and mutant (white) biopsies, normalized by the ratio of input chromatin between wild type and mutant (Amount_norm_). Error bars indicate the standard deviation in triplicate measurements made from individual biopsy pools. All comparisons are between pairs (WT, MUT) of biopsy pools processed in parallel on the same day. If a particular amplicon was examined in several pairs of biopsies, data from the pair with the highest significance of occupancy is shown. *Pitx2-ChIP Analysis.* (**A–B;** **E–F;** **I–J;** **M–N**). The probability that Pitx2 occupies the site in wild type tissue is indicated by asterisk code above the site and the bar graphs of the measurement; ****(>99.99%), ***(>99%), **(>95%), *(>90%), ± (>70%), ne (no evidence), nd (not detected). One code is shown for each extract pair measured. The significance of occupancy indicated by the code was established by the Student’s t-test between triplicate measures taken on mutant and wild type samples. P-values were looked up from computed t-values for a two-tailed test, and indicate the probability that the observed difference (in either direction) is due to measurement error. Note that the size of the bars does not directly indicate occupancy levels for the simple reason that mutants lack Pitx2 protein and therefore *de facto* have zero occupancy. The size of white bars therefore only indicates total noise of measurement, using exactly the same antibody preparation as the parallel wild type measurement, and must be subtracted from that measurement to obtain a direct measure of occupancy (Fig. 8). *HDAC-ChIP Analysis* (**C–D;** **G–H;** **K–L**) Significance of difference was measured and is encoded as for Pitx2 ChIP analysis. In these experiments, the significance code indicates the chance that the HDAC occupancy differs between WT and MUT.

**Figure 7:**
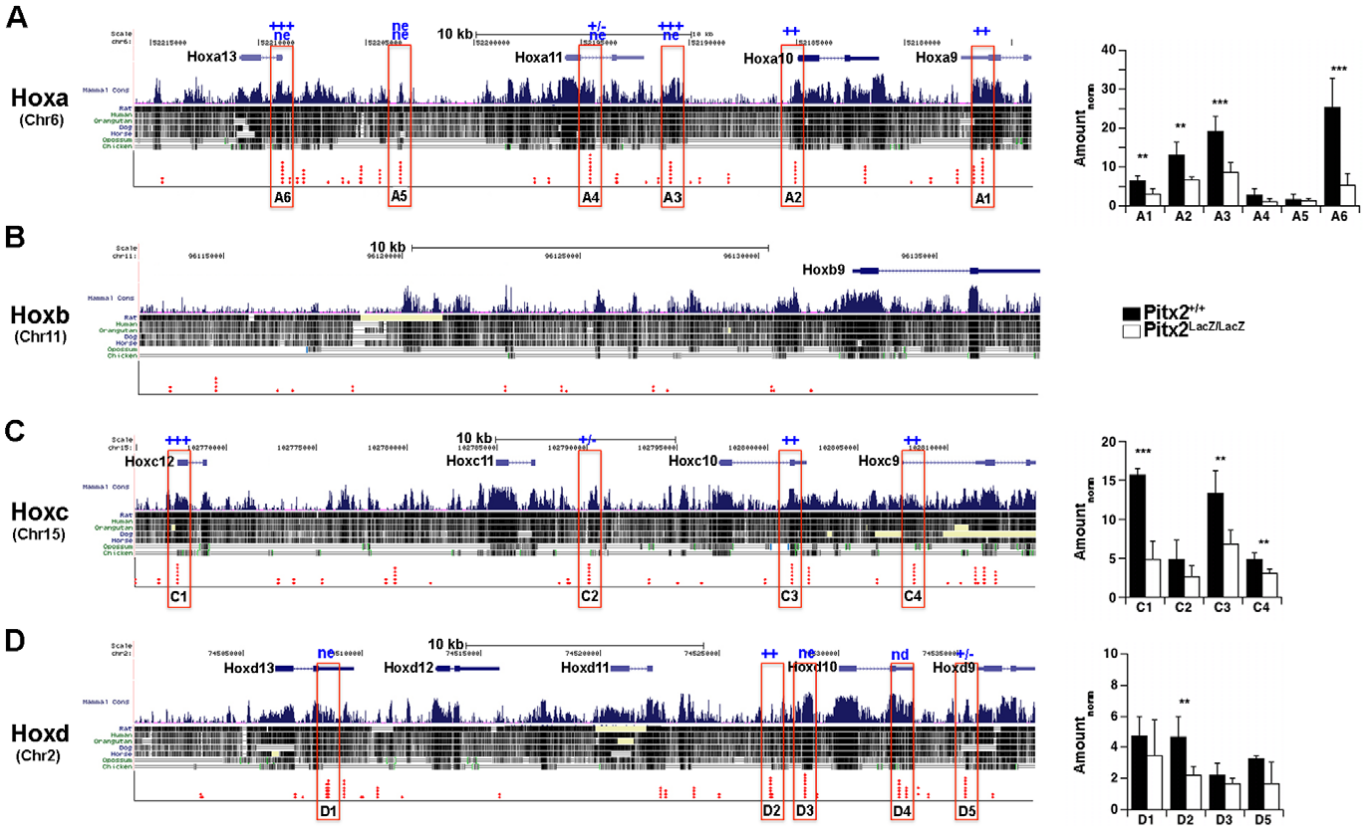
Pitx2 Protein Occupancy at Hox Loci in E10.5 Body Wall Chromatin. The Hox 9-11 paralog regions of the Hoxa (**A**), Hoxb (**B**), Hoxc (**C**), and Hoxd (**D**) clusters were examined for Pitx2 occupancy in sonically sheared chromatin isolated from E10.5 embryonic body wall biopsies. Bar graphs show the average amount of signal precipitated from wild type (black) and mutant (white) biopsies, normalized by the ratio of input chromatin between wild type and mutant (Amount_norm_). The probability that Pitx2 occupies the site in wild type tissue is indicated by asterisk code above the site and the bar graphs of the measurement; ****(>99.99%), ***(>99%), **(>95%), *(>90%), ± (>70%), ne (no evidence), nd (not detected). Error analysis was done as described in the legend to Fig. 6.

Chromatin occupancy analyses provided another means to assess the interaction between Pitx2 and the Hox and MRF gene families *in vivo* at E10.5. Embryos from approximately 3 to 4 synchronous litters were rapidly genotyped by observation in each of 14 separate experiments. Tissue biopsies dissected from 8–11 embryos, for each genotype, were pooled for single experiments. The same type of biopsies was used as for the expression microarray analysis above. Each experiment generated a pair of chromatin extracts, WT and MUT, with sufficient material for one immunoprecipitation with either the anti-Pitx2, anti-HDAC1, or anti-HDAC3 antibodies. Each immunoprecipitate provided enough material for 6–12 triplicate qPCR analyses. A total of 49 triplicate analyses, with different antibody/extract/primer pair combinations provided usable qPCR data. The ratio of WT to MUT input signals, the normalization coefficient, was used to obtain normalized signals for the WT precipitate (WtPPT_Norm_) and thereby insured that the equivalent amounts of chromatin were being compared (Fig. 6, 7). Normalization coefficients were determined by dividing the average signal of three WT replicates by the average signal of three MUT replicates. The normalization coefficients over the entire range of experiments had an average of 1.2±0.7, and ranged from 0.6 to 1.55, indicating that MUT and WT extract pairs never differed more than 1.7 fold in the amount of chromatin that was immunoprecipitated and processed in parallel.

Amplicons within the MRF and Hox loci were identified as described previously for the T-box genes [20]. Core motifs for bicoid class homeodomains (TAATCY) that were embedded in evolutionarily conserved non-coding regions, and were themselves evolutionary conserved, were identified (Figs. 6, 7). Each diamond represents a different species in which the core motif was conserved. Core motifs that were conserved more deeply in evolution were expected to be more essential for biological function. Core motifs with the most diamonds were selected as candidate CRMs, and primers pairs were designed to encompass a 70–150 bp context around these sites. The initially selected primer pairs were tested by endpoint PCR on purified genomic DNA. Amplified pairs were selected for Pitx2, HDAC1, and HDAC3 chromatin occupancy analyses by ChIP-qPCR (Table S1).

**Figure 8:**
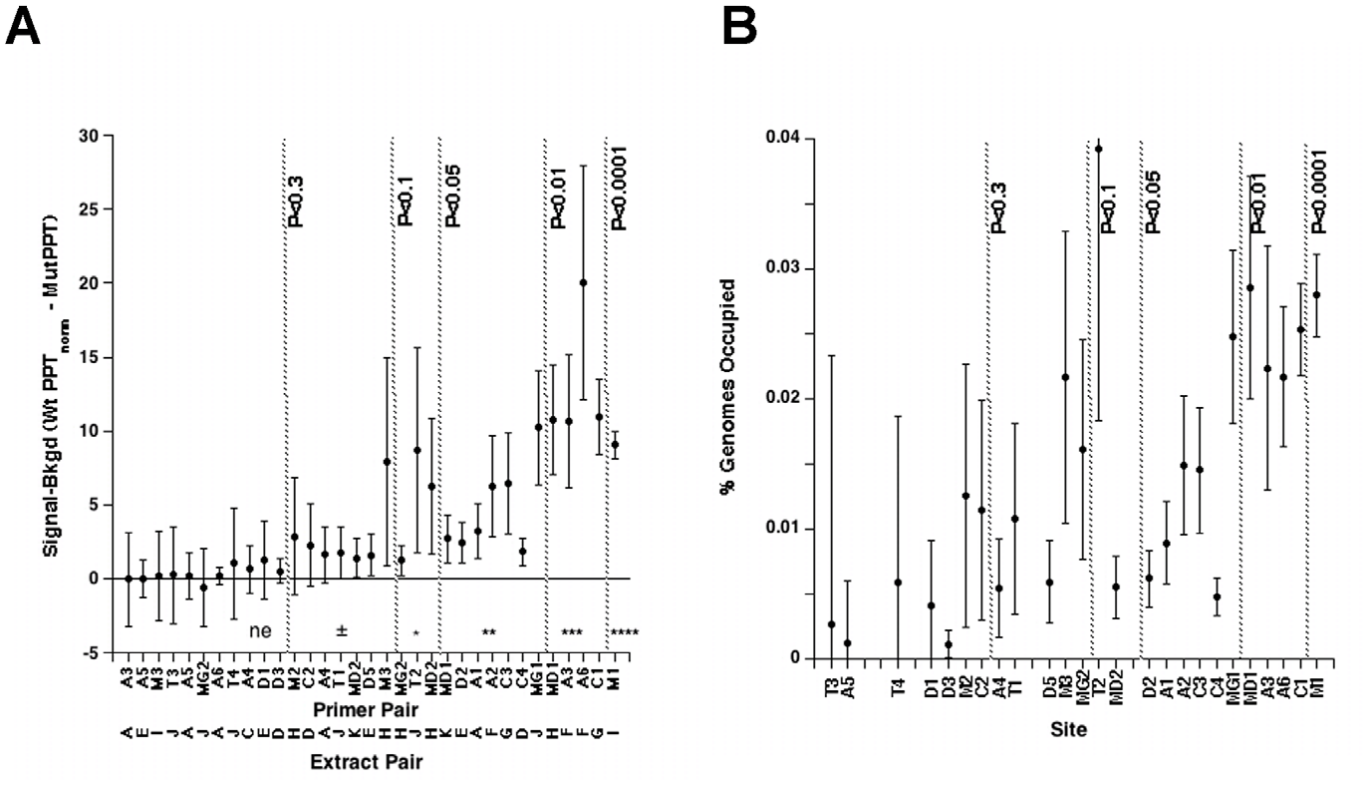
Determination of Absolute Pitx2 Occupancy levels in Embryonic Biopsies. (**A**) Normalized WT chromatin immunoprecipitate signals (WTPPTnorm) were obtained by dividing signals from wild type precipitates by the ratio (WT/MUT) of input signals. Mutant chromatin immunoprecipitate signals (MUTPPT) represent collective measurement noise, with the identical antibody preparation, and were therefore subtracted to obtain the absolute amount of signal due to Pitx2 occupancy, which is plotted on the Y-axis. Error bars were calculated, by standard error propagation techniques, from the standard deviations in the measurements of both the wild type and mutant signals. The primer pair and extract pair used for each measurement is indicated on the X-axis. Data was sorted by significance of occupancy computed as described in legend to Fig. 6. Significance coding thresholds are indicated by vertical dashed lines, with associated P-values. Note that the Pitx2-specific signal approaches zero as the significance of occupancy approaches accepted scientific standards (P<0.05 or P<0.1). (**B**) The percent of genomes in the biopsy that were occupied by Pitx2 was calculated, for each particular primer-pair/extract combination, by dividing the Pitx2-specific signal by the average input signal (wild type input measurements were normalized by the overall input ratio before being averaged together with the mutant input measurements). Error bars were calculated directly from measurement errors by standard error propagation techniques executed at each step in the calculation. Data is sorted in the same order as in panel A. Gaps on the x-axis indicate a primer pair that was tested in more than one extract pair; only the measurement of highest significance is shown. Note that there is an approximate correspondence between the amount of Pitx2-specific signal (Panel A) and the percentage of genomes occupied (Panel B) at low P-values. This correspondence breaks down as P-values rise above accepted scientific norms (P<0.05 or P<0.1). Within the significant zone, the correspondence between absolute Pitx2-specific signal and calculated percent occupancy appears to be determined by the efficiency of particular probe sets in generating signal.

The MUT extract lacks Pitx2 protein and is therefore expected to have 0% occupancy. The signal measured in the MUT precipitate, for any given amplicon, is a direct measurement of the background produced by the immunoprecipitation for that amplicon. The MUT precipitate signal was therefore subtracted from the WT precipitate signal (normalized for overall input) to calculate the signal produced from Pitx2-occupied fragments (Fig. 8A). This difference was positive for 32 out of 33 triplicate assays, suggesting that Pitx2 occupies all the fragments tested at some level. The P value, which indicates the chance that the WT and MUT signals differ by chance alone, is negatively correlated with the magnitude of the difference between wild type and mutant signals. In contrast, the error of measurement (error bars) appears to be similar at all P values. The error bars cross the zero line as P values become greater than 0.1 and these sites are marked as no evidence (ne; Fig. 6, 7). The fact that precipitated signals of WT and MUT extracts are correlated along a line suggests that almost all sites tested show some level of Pitx2 occupancy. The slope of the line indicates that WT precipitates produced, on the average, 2.4 fold more signal than MUT precipitates, regardless of the particular set of sites tested in any given extract. The average input signals of WT and MUT extracts were correlated along a line with slope 1.1 (R = 0.95), showing that the systematically higher signals in WT precipitates were not due to systematically more WT tissue (Fig. 8B). Quantitative comparisons required that both of the compared sites showed significant occupancy in the same extract. In general, the sites that had significant measurable occupancy appeared to have similar absolute occupancy levels. The fraction of genomes occupied in the biopsy was computed for each site by dividing the Pitx2-specific signal (normalized WT precipitate - MUT precipitate) by the normalized input signal (Fig. 8B). This operation normalizes for differential efficiencies in PCR amplifications. The computed percent occupancy (Fig. 8B) generally correlated with the strength of the Pitx2-specific signal (Fig. 8A), suggesting that most amplifications worked with similar efficiency. Both the quality of the extracts and the physical properties of the primers/amplicons appear to noticeably influence signal production efficiency.

### HDAC Occupancy at MRF Sites

Significant Pitx2 occupancy was observed for at least one of the sites tested for each MRF gene. Significant occupancy of the M1 site of Myf5, and Md1 site of Myod1, and Mg1 site of Myogenin were demonstrated in extract I, H and J, respectively. No significant evidence of occupancy was obtained for M2, M3, Md2, and Mg2 in the same three extracts. Pitx2 therefore selectively occupied at least one of the sites selected for each MRF gene. All of the MRFs showed higher expression signals on triplicate arrays when *Pitx2* function was lost. *Pitx2*-dependent repression of Myf5 expression was significant (P = 0.029) and larger fold than repression of the other two MRFs. Chromatin immunoprecipitations with anti-HDAC1 and anti-HDAC3 antibodies were used to determine if corepressors selectively associate with the sites that were selectively occupied by Pitx2 on these genes. No significant increases in HDAC3 occupancy were observed at any of the MRF sites (Fig. 6D, H, L).

HDAC1 showed significant higher occupancy at the M1 site in WT biopsies than in MUT biopsies, indicating that loss of Pitx2 occupancy was associated with the loss of corepressor occupancy at this site (Fig. 6C). The loss of corepressor along the Myf5 locus was not uniform in mutants because no significant difference was observed at the M3 site in the same extract. HDAC1 occupancy at the Md1 and Mg1 sites, which are normally occupied by Pitx2, did not change significantly in mutants (Fig. 6G, K), indicating that Pitx2 occupancy alone does not necessarily increase HDAC1 occupancy levels. The *Pitx2*-dependent HDAC1 occupancy at a Pitx2 occupied site in Myf5 correlates well with the *Pitx2*-dependent repression of Myf5, and indicates that Pitx2 plays a required role in recruiting corepressors at the M1 site. Pitx2 may be similarly required at the Md1 and Mg1 sites, but this requirement may only become measurable at these sites as they come into use during another phase of development.

## Discussion

### Pitx2 Role in Abdominal Wall Development

**Figure 9:**
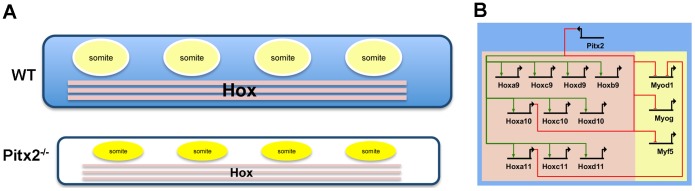
Developmental and Draft Transcriptional Network Models. (**A**) Hypaxial Pitx2^-^ somites (yellow) become embedded in the Pitx2^+^ SMP (blue) prior to the onset of Pitx2 expression in the somites. The SMP expresses an abdominal lateral plate mesoderm *Hox* code (bright pink). Loss of Pitx2 alters the *Hox* code in the abdominal wall (dull pink), but does not affect expression levels of MRFs in somites (still yellow). The altered surroundings stunt the somites and ultimately prevent muscle development in the abdominal wall. (**B**) Draft network model shows that Pitx2 normally activates *Hox9-11* paralogs in their abdominal domains and represses MRFs in somite-derived structures such as the myotome, and dermomyotome in the abdominal wall cells. Interactions of Pitx2 in somatic cells are not depicted in this model.

The abdominal walls of *Pitx2* mutant mice do not fuse, and abdominal muscles do not form within them. At the anatomical level, it is easier to explain the abdominal muscle deficits of *Pitx2* mutants as secondary consequences of SMP defects, than by invoking abdomen-specific regulatory circuits for the muscle lineage itself. The expression domains of Pax3 and MRFs, which play key roles in specification, determination and commitment, appear to be normally initiated and maintained in the abdominal somites that give rise to the abdominal muscles. Moreover, Pitx2 is not expressed in the myotome or dermomyotome until a later stage, and its expression is neither restricted to abdominal somites along the anterior-posterior axis nor to the abdominal extensions of the somites in the dorsal-ventral axis. A simpler model (Fig. 9A) can be developed by noting that Pitx2 is robustly expressed in an axially-restricted, and ventrally-restricted fashion in abdominal LPM, in an expression domain that begins in early gastrulation along with those of the *Hox* and T-box genes. The genetic regulatory interactions of *Pitx2* with both T-box [20] and *Hox* genes (this report) may therefore become established as network kernels well before E10.5.

The severe deformities of the body wall begin well before the hypaxial extensions of the abdominal somites are formed and are therefore unlikely to be due to secondary consequences of somite deficiencies per se. They are far more likely to result from misspecification of the abdominal LPM early in gastrulation. This code is apparently defective in the abdominal SMP of Pitx2 mutants and does not support appropriate muscle development. Pitx2 also exerts a strong influence on jaw development. The first BA, from which the jaw derives, is also a hypaxial structure filled with mesenchyme derived from the LPM. The Pitx2 expression domain in this zone begins during early gastrulation and loss of Pitx2 causes reduced and altered growth of the first BA prior to loss of muscle [9]. The striking parallels suggest that Pitx2 normally contributes to the specification of cellular states in the LPM at two specific axial levels, representing two distinct network kernels. Pitx2 is very likely to play a significant role in the myogenic lineage, but that role is different from specification, determination, or commitment. The phenotype associated with the myogenic role of Pitx2 is not loss or displacement of muscle. It is the alteration of texture that is associated with all muscle anlagen in the Pitx2 mutant, even at locations distant from the gastrulation-associated expression domains of Pitx2.

### Pitx2-dependent Regulation of Hox 9-11 Paralogs in Lateral Plate and Intermediate Mesoderm

Our data indicate that *Pitx2* consistently upregulates Hox genes of the 9, 10, 11 paralog groups in abdominal biopsies taken at E10.5, while having few significant effects on Hox genes in other paralog groups. The Hox 9, 10, and 11 paralogs generally have anterior expression boundaries and phenotypes in the abdominal region, where Pitx2 is expressed and where *Pitx2* mutants also have phenotypes [35]–[37]. *Hox* 9, 10, and 11-dependent axial phenotypes are described in terms of vertebral morphology [38], [39] or neural tube patterning [40], [41], and arise from changes in the nested expression patterns, or *Hox* codes [18] in the paraxial mesoderm and neuroectoderm, respectively.

*Hox* expression begins during gastrulation, as ingressing cells form mesoderm, well before the formation of presomitic mesoderm and gradually sharpens to form anterior boundaries in the presomitic mesoderm [42], [43]. The Hox genes are not robustly expressed in the more exterior compartments of the maturing somite, the myotome and dermomyotome [44]. The mesodermal signal observed in Hox whole mount *in situ* hybridizations diminishes as vertebrae mature and become more internal [32]. At E10.5, the anterior boundary of somitic expression of Hox 9 paralogs has been marked at somite 23, which is just in front of the hindlimb bud [32], [39]. The anterior boundaries of Hox10 and 11 paralogs in the paraxial mesoderm are more posterior [33], [45]. Pitx2 protein only just begins to be expressed in somites at the posterior margin of forelimb buds at E10.5. Mutations of Hox genes often lead to defects in vertebrae and ribs, which are sclerotome derivatives. Few, if any, Hox phenotypes have been discussed in terms of muscle specification. It is therefore unlikely that Pitx2, which is expressed later, in the dermomyotome/myotome compartments of the somite, and at more anterior levels, exerts positive regulatory influence over the Hox9-11 paralogs. The *Pitx2*-dependent *Hox* expression that we observe is therefore unlikely to occur in the paraxial mesoderm portion of our biopsies.

The anterior expression boundaries for individual Hox genes occur at different axial levels in the ectoderm, paraxial mesoderm and lateral plate mesoderm. The Hox9 paralogs are expressed in the interlimb LPM and have well characterized anterior expression boundaries that are close to the posterior margin of the forelimb in both chick [46] and mice [32], [39]. The expression and boundaries of Hox10 and 11 in the LPM have, to our knowledge, not yet been reported. Hoxa9, b9, a10 and c10 were expressed in the interlimb flank and Hoxa9, c10, d10, a11, and d11 were expressed in an elongated structure at the position where the kidney develops from the intermediate mesoderm. Hox9 gene expression in LPM has been implicated in positioning of limb bud outgrowth [46] and in early anterior-posterior patterning of the forelimb [47], whereas Hox10 [48], Hox11 [38] have been implicated in intermediate mesoderm/kidney development. Pitx2 has been also implicated in kidney development [49], suggesting that it may also influence the intermediate mesoderm. The *Pitx2*-dependent regulation of Hox9, 10, and 11 genes therefore occurs in either LPM or intermediate mesoderm derivatives.

The body wall clearly behaves aberrantly in the *Pitx2* mutant. The principle of posterior dominance would suggest that the purpose of Hox9 and Hox10 expression is to suppress a more anterior, or earlier, Hox code in the LPM. The LPM just anterior to the flank normally gives rise to the forelimb bud. The Pitx2 mutant body wall grows outward rather than inward, but we do not observe an ectopic limb bud emerging from the flank. Formation of limb bud requires induction of FGF10 [50] and FGF8/FGF4 [51] by cues that are thought to come from the adjacent intermediate mesoderm. The abdominal LPM of mutants is not adjacent to the limb bud inducing signals and therefore we do not expect ectopic limb buds to form. The levels of the three critical FGFs are unaffected by the loss of Pitx2 in our array measurements (data not shown), consistent with the lack of an ectopic limb bud. Ventral muscle progenitors at limb levels normally become migratory, but the abdominal somite extensions in *Pitx2* mutants remain intact, indicating that they do not adopt the limb level characteristics as they come into contact with the respecified LPM. However, delamination requires SF/HGF signaling from the limb bud mesenchyme, which does not form [52] and requires paraxial *Hox* specification [53]. The lack of limb buds and migratory muscle precursors therefore does not exclude the idea that abdominal LPM is re-specified to an earlier/more anterior form of LPM that does not support abdominal muscle extensions. We have previously shown that Pitx2 also exerts significant regulatory control over the T-box genes in the abdominal wall at this stage [20], [54]. It represses Tbx5, which marks LPM of the presumptive forelimb, and activates Tbx4, which marks the LPM of the presumptive hindlimb. Axial specification of the LPM involves both T-box and Hox genes and appears to be important in positioning hypaxial structures (limbs, jaws) in correct relationships with axial structures [55].

### Pitx2-dependent Regulation of Myf5

*Pitx2*-dependent repression of Myf5 was observed in arrays and by qPCR, and *Pitx2*-dependent HDAC1 recruitment was observed at the Pitx2-occupied M1 site between the Myf5 and Myf6 genes. The SMP and SPP play host to developing MRF^+^ muscle anlagen, but lack MRF expression themselves. The M1 element on the Myf5 gene may be used to insure that Myf5 expression stays off in cells that surround the muscle anlagen. A similar logic appears to apply to other genes that distinctly mark structures, such as ribs and muscles that develop within the body wall (Table 1). The other MRFs are all slightly repressed by Pitx2 in array analysis [56]. Pdgfc [57] specifically marks the myotome and was repressed. Markers of the hypaxial sclerotome, such as scleraxis, Pax1, and tenascin C are also repressed. Consequently, Pitx2 protein in abdominal LPM cells appears to suppress genes that are expressed in the cells of adjoining structures. Pitx2 therefore seems to be defining, or specifying, the body wall mesenchymal cell type by insuring that it does not become any other, neighboring cell. This is similar to the situation we have described for Lbx1 in the neural tube [58].

### Draft Regulatory Network

The process of establishing a transcriptional regulatory network can be considerably accelerated if Pitx2-dependent effects on gene expression are measured on a genome wide basis in embryo biopsies using expression microarrays. While this approach gives many more gene expression changes than one can deal with at once, these changes are generally real and biologically significant when interpreted in light of available expression patterns. The myotome, dermomyotomes, and LPM are each likely to be composed of several cellular states, for which the same argument holds. We have now demonstrated that Pitx2 regulates and Pitx2 occupies sites in, T-box genes, Hox genes, and MRF genes in the abdominal wall biopsies. More experiments will be required to establish in which Pitx2-expressing populations of the biopsy these genetic and physical interactions occur. This will allow the draft “view from the genome” model (Fig. 9B) to be converted to a “view from the cell” model [16], [17]. This can be more feasible by using tissue specific mouse models. The ocular and umbilical deficiencies observed in the autosomal dominant disorder Axenfeld-Rieger syndrome associated with Pitx2 mutations [59] further supports the idea of the involvement of Pitx2 in different network kernels.

## Materials and Methods

### Mice

All research was conducted according to the protocols reviewed and approved by the Oregon State University Institutional Animal Care and Use Committee. The Pitx2^+/LacZ^ mouse line was maintained on an outcrossed ICR background. Noon on the day of a vaginal plug was considered embryonic day (E) 0.5. Yolk sacs of embryos were used for genotyping.

### Microarray Analysis

Total RNA was prepared using Qiagen RNeasy Mini kit. Microarray probes were created using Affymetrix one-step labeling, and used to probe the Affymetrix Mouse Genome 430 2.0 gene expression array. Array results have been deposited for public access at ArrayExpress under the accession number E-MEXP-2332. The raw*.cel files were normalized by RMA using RMAExpress, and data was analyzed using conventional spreadsheet (Excel), graphing (Kaleidagraph), and relational database (Filemaker Pro) software. Fold scanning analysis was used to determine the false discovery rate as a function of fold cutoff [58].

### X-gal Staining, Whole Mount In Situ Hybridization, Immunohistochemistry

The X-gal staining procedure was as previously described [9]. Embryos for whole mount in situ hybridization were fixed overnight at 4°C in 4% paraformaldehyde/0.1 M NaPO_4_ pH 7.4 and washed twice for 5 min in phosphate buffered saline (PBS) containing 0.1% Tween 20 (1xPBST). Embryos were dehydrated, 5 min each in 25%, 50%, and 75% methanol (diluted with 1xPBST) and twice for 5 min in 100% methanol, before being stored at −20°C in 100% methanol. Embryos were rehydrated as needed by reversing the methanol series for 5 min each in 75%, 50%, and 25% methanol before washing twice for 5 min with 1xPBST. Embryos were bleached in freshly prepared 6% H_2_O_2_/1xPBST for 1 h, washed 3 times 5 min in 1xPBST, and permeabilized by proteinase K treatment at room temperature (RT). A frozen Proteinase K stock (10 mg/ml) was diluted 1∶500 in 1xPBST and applied until embryos were transparent by visual inspection. This takes approximately 20 min for E10.5 and 25 min for E11.5 mice. Glycine was added to 2 mg/ml, from a 40 mg/ml stock, to stop the proteinase reaction and embryos were washed twice for 5 min in 1xPBST prior to fixing in 4% paraformaldehyde/0.1% glutaraldehyde (from frozen 25% stock)/1xPBST. Fixative was removed by two 5 min 1xPBST washes before embryos were prehybridized at 65°C for 1 h in hybridization buffer (50% deionized formamide, 5XSSC, 0.1%SDS, 50 µg/ml Heparin, 0.2 mg/ml yeast tRNA). Denatured DIG probes were added for 16–18 hours at 65°C with agitation. Embryos were transferred to 6-well dishes, washed 3 times 20 min with pre-warmed prehybridization buffer at 65°C, washed twice for 30 min at RT with MABT (333 mM NaCl, 2% blocking solution, 0.5% Tween20; 100 mM maleic acid pH7.0), treated 1 h with 100 µg/ml RNaseA at 37°C in RNase buffer (0.5 M NaCl, 10 mM TisHCL pH7.5, 1 mM EDTA), washed 3 times 5 min with MABT, blocked 1 h in 10% heat inactivated horse serum in MABT, and incubated overnight at 4°C with anti-DIG antibody (1∶2,000). Embryos were washed 8–16 times over the course of 24–48 h with MABT and three times 10 min at RT with color reaction buffer (0.1 M NaCl, 50 mM MgCl_2_, 0.1 MTris-HCl pH 9.5, 0.1% Tween20, 2 mM levamisole) before starting the color reaction by adding NBT to 0.03% and BCIP to 0.015%. Color reactions were stopped with 1xPBST. A Discovery V8 Zeiss microscope with an Axiocam system was used to photograph the processed embryos. Wild type and mutant embryos were processed and IHC was performed on 14 µm sections as previously described [9]. Primary antibodies listed as follows: ß-galactosidase (Rabbit, 1∶1000, Cappel), Myog (mouse, 1∶100, Pharmagen).

### Quantitative Real - time PCR (qPCR)

cDNA or Immunoprecipitated (IP) DNA from wild type and Pitx2 mutant mice were analyzed by qPCR on the ABI 7500 machine using SYBR Green 1 methodology as previously described [20]. Samples were run in technical triplicates from pooled tissue preparations from 3–4 E10.5 Pitx2 litters containing on average 16 embryos each. Expression analysis was normalized against glyceraldehyde-3-phosphate dehydrogenate expression levels, while IPs were normalized against input. All primers were tested for specificity with standard PCR and indicated in Table S1.

### Pitx2 Binding Site Analysis

The absolute location and evolutionary conservation of potential Pitx2 binding sites, with consensus sequence TAATCY, was identified by the use of an in house Perl script, Binding_site_compare.pl [54], [60], [61]. Individual gene alignments, along with the −20 kb upstream region were downloaded from the UCSC Genome Browser on Mouse July 2007 (NCBI37/mm9) Assembly available at http://genome.ucsc.edu/, and formatted for our script. The script concatenated the alignments from the UCSC Genome Browser, identified the absolute Pitx2 binding site locations for each gene based on the mouse sequence, and reported the species for which each binding site was conserved within. Excel was used to map binding site locations to each gene cluster.

### Pitx2/HDAC Occupancy Validation by ChIP (Chromatin Immuno-Precipitation)

Abdominal wall tissues from 8–11 embryos, pooled from 3–4 litters of E10.5 Pitx2 WT and MUT mice were harvested per ChIP. Samples were processed as previously described [20]. Small portions of the extract pairs were compared by electrophoresis to confirm that the size distributions of chromatin fragments were similar. The size distribution of sheared fragments was typically between 200 and 500 bp, and was centered at approximately 300 bp. Further small portions of each extract pair, the input fractions, were subjected to qPCR in parallel to their precipitated counterparts to determine the relative proportions of genomes in each extract pair. Primers were designed for binding sites identified as conserved within a minimum of 6 species. Control primers were designed for regions on the genome with no putative binding site within a minimum of a 1 kb window on the mouse genome. All ChIP-qPCRs were performed in technical triplicates. Threshold cycles (C_t_ values) were taken as output from the qPCR software and processed further in a relational database (FilemakerPro). C_t_ values should not be averaged directly because they are logarithmic in nature. They were therefore converted to signals (arbitrary units) by the equation (signal  = 10^10^ E^−Ct^), where E is the PCR efficiency (between 1 and 2).

## Supporting Information

Figure S1 **Quantitative analysis of MRFs in abdominal wall biopsies.** RNA qPCR analysis of abdominal wall biopsies from WT and MUT E10.5 mice, by using specific primers for Myod1, Myf5 and Myogenin. The relative abundance was calculated and RNA levels were higher in MUT biopsies in accordance to microarray expression levels in Table1.
(TIF)

Table S1 (PDF)
