# Correction: Loss of Abdominal Muscle in *Pitx2* Mutants Associated with Altered Axial Specification of Lateral Plate Mesoderm

**DOI:** 10.1371/annotation/c980d5a1-1899-4d30-af7b-573d4049cf4b

**Published:** 2012-10-17

**Authors:** Diana Eng, Hsiao-Yen Ma, Jun Xu, Hung-Ping Shih, Michael K. Gross, Chrissa Kiouss

The sixth author's name was spelled incorrectly. The correct name is: Chrissa Kioussi.

