# Supplementary figures and images for: Loss of Abdominal Muscle in Pitx2 Mutants Associated with Altered Axial Specification of Lateral Plate Mesoderm
Source: PLoS One. 2012 Jul 31;7(7):e42228. doi: 10.1371/journal.pone.0042228 (PMC3409154; doi:10.1371/journal.pone.0042228)

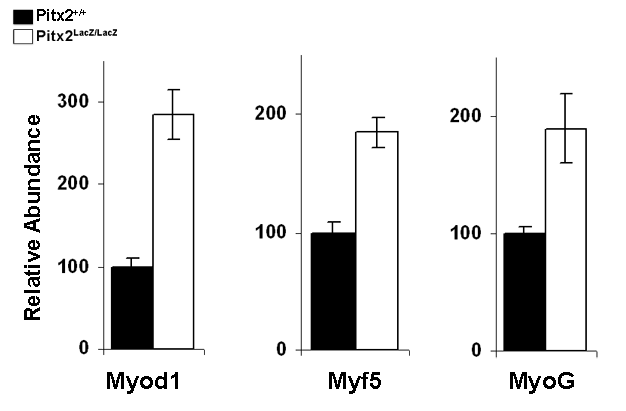

Supplement: Figure S1 — Quantitative analysis of MRFs in abdominal wall biopsies. RNA qPCR analysis of abdominal wall biopsies from WT and MUT E10.5 mice, by using specific primers for Myod1, Myf5 and Myogenin. The relative abundance was calculated and RNA levels were higher in MUT biopsies in accordance to microarray expression levels in Table1. (TIF) [file pone.0042228.s001.tif]
