# Supplementary material for: Loss of Abdominal Muscle in Pitx2 Mutants Associated with Altered Axial Specification of Lateral Plate Mesoderm
Source: PLoS One. 2012 Jul 31;7(7):e42228. doi: 10.1371/journal.pone.0042228 (PMC3409154; doi:10.1371/journal.pone.0042228)
Supplement: Table S1 — (PDF) [file pone.0042228.s002.pdf]

Supplement Table 1

Primer	Forward	Reverse
**qPCR**		
Hoxa9	CCCCGACTTCAGTCCTTGC	GATGCACGTAGGGGTGGTG
Hoxa10	CCTGCCGCGAACTCCTTTT	GGCGCTTCATTACGCTTGC
Hoxa11	TTTGATGAGCGTGGTCCCTG	AGGAGTAGGAGTATGTCATTGGG
Hoxb9	GCACGCCCGAGTACAGTTT	GGCAGAGGGGTTGGTTTGA
Hoxc9	ACTCGCTCATCTCTCACGACA	AGGACGGAAAATCGCTACAGT
Hoxc10	ATGACATGCCCTCGCAATGTA	CCCCGCAGTTGAAGTCACTC
Hoxc11	TTTGATGAGCGTGGTCCCTG	AGGAGTAGGAGTATGTCATTGGG
Hoxd9	CAGCAGCAACTTGACCCAAAC	TCCAGCTCTAGCGTCTGGTAT
Hoxd10	ACCTATGGAATGCAAACCTGTG	TCTGTCCAACTGTCTACTTGAGG
Hoxd11	AAAAGACTCCAACTCTCTCGGA	AGACGGTCCCTGTTCAGTTTC
Myod1	CGGGACATAGACTTGACAGGC	TCGAAACACGGGTCATCATAGA
Myf5	TGCCAGTTCTCCCCTTCTGA	GGGATACAGGAGCCTTCATAGA
Myog	ATGGAGCTGTATGAGACATCCC	TACACACCTTACATGCCCACG
GAPDH	AGGTCGGTGTGAACGGATTTG	TGTAGACCATGTAGTTGAGGTCA
**ChIP-qPCR**		
A1	GACCCGCGCCTTCCTGATGG	TGCAAAGGGATCGCGCAGCT
A2	CGAGCCAGAGTTTCCACGCG	CCATAGGTGTCAGGCGTGCGTC
A3	GCGGGCAGGGAAGCTCGGTTTA	GCTCAGAAATCAAACCCGCGCCC
A4	CGGAGGTGGGCGCCTGTAAAT	TCCCGCCGCAACACATGGC
A5	GCAAAACCACCTTCCACGCAGC	CTCCCCGGGCTCCAGGCATTA
A6	GCAAACGTCAGCGTCTCTCTATCCA	GAGGCAGGTCACAATCTGGTTCCA
C1	GCGGTAGCGCCCACGTGAGTA	TATTCACCAGCGGCCCCACAAAC
C2	GCACTCTGGGCTCAACCCCTTG	AGGGACGCTTTATGGCGGCGT
C3	TATTTGACGCGAGAGCGCCGC	TGGAGGTCAGTTCCCGGATCCG
C4	CCCGCTGGGGGCACAGAAG	CTCCCCCTACCGCGTGTCTC
D1	CCCCCAAATGAATTTCAGAAG	AGCTGTTTCCACTTGCCTTG
D2	GCCTCTCCCACTTCACTGCAGA	AATCCCAAGCAATGCAGAGCTGTTC
D3	ACCTTCTTGGTTAGTAGCGGAGGGG	CCGCTGTTGCCACAAACGGG
D5	CGAGCTGCTCCATCCGTCCG	CACACAGCTTGGCGTGTTGTTT
Ctrl2	AGGGCCCTAAGACGTTGGTGGG	GCCGTGGAGCCCTTGGTACG
Ctrl4	GGCCTCAGGTCCCACAGGGTAA	TAGCACCTGAGCAGCCGAAAGC
Ctrl 6	CCCTCTGAGAAGCGCTACCACAGA	CCCTCCTATGCCTGTCGGCG
M1	TTTTGGCTTTGACCTTGGTT	TGGAGTGAGGTTGCCTATGA
M2	CCTGCTAACCAGGCAACTTC	TGGCTATTGCCTATGAAATCTCT
M3	TCCTAGCATGAGCAACAACTG	CCCTCATGTTCATGGAGAAGT
Md1	TCTAGGGCAGCTGCTGTGGGC	GTCAGTTCATCTGCCCTCCTGGGG
Md2	CCCCACTCTCCCCCTTCCCG	TCCCGCTCTGGCCAAGCAAC
Mg1	GTGGTGGTGACGCTGGACGG	TGTGGGAGGGACAGGTCACCG
Mg2	GCATGACCGCCTGAGGAGGC	ACCTGACAGCAGGGCGTCCA
Mg3	GACCCGTGCACAGGAGCCG	CGCCACAGAAACCTGAGCCCC
